# Topical inflammasome inhibition with disulfiram prevents irritant contact dermatitis

**DOI:** 10.1002/clt2.12045

**Published:** 2021-07-22

**Authors:** Hanna Bonnekoh, Carolina Vera, Angela Abad‐Perez, Silke Radetzki, Martin Neuenschwander, Edgar Specker, Niklas Amadeus Mahnke, Stefan Frischbutter, Eicke Latz, Marc Nazaré, Jens v. Kries, Marcus Maurer, Jörg Scheffel, Karoline Krause

**Affiliations:** ^1^ Dermatological Allergology, Allergie‐Centrum‐Charité, Department of Dermatology, Venereology and Allergology Charité – Universitätsmedizin Berlin, Corporate Member of Freie Universität Berlin, Humboldt‐Universität zu Berlin, and Berlin Institute of Health Berlin Germany; ^2^ Autoinflammation Reference Center Charité (ARC2) Charité – Universitätsmedizin Berlin Berlin Germany; ^3^ Department of Chemical Biology Leibniz‐Forschungsinstitut für Molekulare Pharmakologie (FMP) Berlin Germany; ^4^ Institute of Innate Immunity University of Bonn Bonn Germany; ^5^ German Center of Neurodegenerative Diseases (DZNE) University of Bonn Bonn Germany

**Keywords:** autoinflammation, contact dermatitis, disulfiram, inflammasome, interleukin‐18

## Abstract

**Background:**

The pathogenesis of contact dermatitis, a common inflammatory skin disease with limited treatment options, is held to be driven by inflammasome activation induced by allergens and irritants. We here aim to identify inflammasome‐targeting treatment strategies for irritant contact dermatitis.

**Methods:**

A high content screen with 41,184 small molecules was performed using fluorescent Apoptosis associated speck‐like protein containing a CARD (ASC) speck formation as a readout for inflammasome activation. Hit compounds were validated for inhibition of interleukin (IL)‐1β secretion. Of these, the approved thiuramdisulfide derivative disulfiram was selected and tested in a patch test model of irritant contact dermatitis in 25 healthy volunteers. Topical application of disulfiram, mometasone or vehicle was followed by application of sodiumdodecylsulfate (SDS) for 24 h each. Eczema induction was quantified by mexameter and laser speckle imaging. Corneocyte sampling of lesional skin was performed to assess inflammasome‐mediated cytokines IL‐1β and IL‐18.

**Results:**

Disulfiram induced a dose‐dependent inhibition of ASC speck formation and IL‐1β release in cellular assays in vitro. In vivo, treatment with disulfiram, but not with vehicle and less mometasone, inhibited SDS‐induced eczema. This was demonstrated by significantly lower erythema and total perfusion values assessed by mexameter and laser speckle imaging for disulfiram compared to vehicle (*p* < 0.001) and/or mometasone (*p* < 0.001). Also, corneocyte IL‐18 levels were significantly reduced after application of disulfiram compared to vehicle (*p* < 0.001).

**Conclusion:**

We show that disulfiram is a dose‐dependent inhibitor of inflammasome pathway activation in vitro and inhibitor of SDS‐induced eczema in vivo. Topical application of disulfiram represents a potential treatment option for irritant contact dermatitis.

## 1 INTRODUCTION

Contact dermatitis is a common chronic inflammatory skin disease presenting with erythema, papules, vesicles and scaling at the site of skin contact with irritants or allergens. Its prevalence has markedly increased, causing high annual societal costs and considerable quality of life impairment.^1^ In Europe, about 15% of the general population suffers from contact dermatitis.^2^

Current therapeutic strategies in contact dermatitis include the avoidance of relevant irritants and allergens, the use of emollients, and treatment with topical glucocorticosteroids, UV‐light, or systemic retinoid treatment in severe disease.^3^ In many patients, these treatments are not satisfactory and often come with side effects. Topical glucocorticosteroids as the standard treatment are often associated with skin atrophy and immunosuppression, loss of efficacy over time and insufficient response. Novel and better treatment options for patients with contact dermatitis are needed.

Chronic hand eczema is the main clinical phenotype of both irritant and allergic contact dermatitis. It represents the most frequent occupational disease in Western countries and negatively affects patients’ ability to work, career development and social status. Chronic hand eczema causes significant individual and economic burden. This includes high rates of depression, anxiety and suicidal ideation compared to the general population.^4^ The economic burden originates from sick leave because of the condition, the need to change the workplace or profession and unemployment.^5^ Of note, the intensive hand hygiene during the COVID‐19 pandemic has already increased the incidence of chronic hand eczema.^6^

Allergic contact dermatitis is a T cell‐driven, delayed‐type IV hypersensitivity reaction to allergens that requires prior sensitization of a susceptible individual to the respective allergen. Most exogenous substances such as chemicals or metals function as haptens that may become full allergens only by binding to protein carriers in the skin. Innate immune sensing followed by an inflammatory milieu induces the migration of dendritic cells to lymphoid organs and presentation of hapten‐protein complexes to naïve T cells. Following sensitization, subsequent re‐exposure to the hapten leads to activation and influx of inflammatory cells such as hapten‐specific effector and memory T cells as well as neutrophils promoting the activation of skin mast cells. Cytotoxic CD8+ T cells kill haptenized keratinocytes and induce via mobilization of further inflammatory cells the formation of eczematous skin lesions.^7^ In contrast, irritant contact dermatitis occurs via a direct toxic effect on epidermal corneocytes and keratinocytes by an irritant (e.g., chemical) with subsequent barrier disruption and inflammation without involvement of adaptive immune mechanisms. Irritants may trigger cell necrosis and the release of stress signals (e.g., reactive oxygen species, ATP) and damage‐associated molecular patterns (e.g., heat‐shock proteins) which are sensed by receptors of innate immune cells.^7^ The pathogenesis of both, allergic and irritant contact dermatitis is held to be driven, at least in part, by the activation of the NLR family pyrin domain containing 3 (NLRP3) inflammasome, an innate immune sensor and multimeric protein complex that initiates an inflammatory form of cell death and triggers the release of the proinflammatory cytokines IL‐1β and IL‐18. Contact allergens such as nickel, trinitro‐chlorobenzene, chromium and latex as well as the skin irritant sodium dodecyl sulfate (SDS) induce NLRP3 inflammasome‐mediated IL‐1β secretion in keratinocytes and macrophages.^8^, ^9^, ^10^, ^11^ Furthermore, inflammasome‐deficient mice display impaired early phase reactions during contact allergen challenge.^8^ Inflammasome‐mediated cytokines induce the activation of dendritic and Langerhans cells, endothelial cells and in case of allergic contact dermatitis the recruitment of antigen‐specific T‐cells. Thus, inflammasome pathway inhibition may be an effective strategy for the treatment of allergic and irritant contact dermatitis.

The development of novel therapies is a major unmet medical need in contact dermatitis and inhibition of the NLRP3‐inflammasome pathway is a promising approach. In search for inhibitors of the NLRP3 inflammasome, we performed a high content screen with 41,184 small molecules by using a recently established reporter cell line expressing a fluorescently tagged apoptosis associated speck‐like protein containing a CARD (ASC).^12^ Here, we report the identification of disulfiram, a known carbamate derivative, to be a dose‐dependent inhibitor of inflammasome activation in vitro and inhibitor of eczema in a patch test model of irritant contact dermatitis in vivo.

## 2 METHODS

### 2.1 High content screen and selectivity screens for inflammasome inhibitors in murine cells

High content screening was performed with 41,184 small molecules including drugs from Selleck, World Drug Index (WDI) derived molecules,^13^ the Library of pharmacologically active compounds (LOPAC, Sigma Aldrich) and non‐commercial compounds from academia. For the high content screen we used an immortalized murine macrophage reporter cell line expressing a fluorescently tagged ASC (ASC‐mCerulean) that we recently established.^12^ At inactive state, the inflammasome adapter molecule ASC is expressed as a soluble cytoplasmatic protein. Upon inflammasome stimulation by activators such as adenosine triphosphate (ATP) or nigericin, ASC assembles into a detectable cytosolic multi‐protein complex named ASC “speck”. The ASC speck formation represents an indicator of inflammasome activation as it correlates with the subsequent cleavage of caspase‐1 and IL‐1β production. All compounds including the previously reported inflammasome inhibitor MCC950^14^ as positive control and vehicle (DMSO) as negative control, were screened for activity at 10 μM concentration for inhibition of ATP‐induced ASC speck formation in the ASC‐mCerulean reporter cell line using a fully automated microscope (ArrayScan, Cellomics Inc.). For details on the high‐throughput screening procedure and data analysis, please refer to Supporting Information S1.^39^ In short, active candidates from primary screening were confirmed in a second, independent round of screening, in which also the selectivity for different types of inflammasome activation was investigated: either using ATP (original screening condition), ATP plus lipopolysaccharides (LPS), or nigericin for inflammasome activation. Furthermore, the effect on IL‐1β production was assessed using an ELISA assay. Compounds with significant inhibition in all conditions were selected for determination of the half maximal inhibitory concentration (IC50) and viability testing at concentrations ranging from 20 μM down to 0.02 μM in the murine reporter cell line (for LPS and ATP‐induced inflammasome activation, measurement in triplicates). The substances were finally selected as hit candidates on the basis of their IC50 dose response profile, chemical structure and potential for chemical optimization and cell count stability (Supporting Information S1).

### 2.2 IC50 determination in human THP1 reporter cells and cytokine responses in human peripheral blood monocytes

IC50 values of hit compounds were also assessed for the human system using THP1 ASC‐GFP reporter monocytes (Invivogen®) analog to the murine reporter cell screening and IC50 delineation process (Supporting Information S1). Additionally, inhibition of IL‐1β and IL‐18 secretion from human peripheral blood monocytes was evaluated. To obtain the monocytes, peripheral blood mononuclear cells from human venous blood were harvested using density gradient centrifugation and seeded into adhesion cell culture plates containing RPMI 1640 (Biochrom) with 10% FBS, 1% penicillin/streptomycin and 50 mg/ml Normocin® (Invivogen). After 2 h of incubation (37°C, 5% CO_2_), the medium containing non‐adherent cells was removed and discarded. The remaining adherent cells (primary monocytes) were carefully washed with medium and used for further experiments. After overnight priming with LPS, cells were stimulated with hit compounds for 1 h, followed by inflammasome activation with ATP. IL‐1β in supernatant was measured by ELISA. IL‐18 secretion by human monocytes was measured using a bead‐based multiplex cytokine assay (for details, please refer to the Supporting Information S2).

### 2.3 In vivo studies in healthy volunteers and study design

For inclusion, the participants had to be ≥ 18 years and had to be able to read, understand and to be willing to sign the informed consent form and abide with study procedures. Exclusion criteria included skin diseases such as psoriasis or infectious skin diseases (viral, bacterial, fungal infections), ongoing treatment with immunosuppressive drugs or phototherapy, an immunosuppressive condition, pregnancy, breast feeding and a known intolerance to mometasone furoate, disulfiram or base cream. The study was approved by the local ethics committee (EA1/160/17), and healthy subjects provided written and oral informed consent before any study‐related procedures (for details, please refer to the Supporting Information S3).

In the first part of the study as proof of concept, three different concentrations of disulfiram and vehicle were applied on the skin of the inner forearm of 16 healthy volunteers using the Curatest® patches (Rengsdorf). After 24 h, patches were removed, and each treated area was incubated with SDS 5% (2 drops) and the test patch was applied for additional 24 h. After further 48 h, the clinical response was assessed (Figure S1A).

The second, double‐blind and placebo‐controlled part of the study was conducted in 25 healthy subjects and consisted of four visits on four consecutive days. On day 1, (i) 5% disulfiram in base cream, (ii) mometasone furoate 0.1% in base cream and (iii) base cream (vehicle) were applied on non‐irritated intact skin of the inner forearm using the patches as before. After 24 h of incubation, the patch tests were removed and SDS 5% (2 drops) was applied on all three treated skin areas covered by patches for 24 h. On day 3, the patches were removed and on day 4 the clinical responses were assessed (Supporting Information S1 and Figure S1B).

### 2.4 Assessment of erythema and skin blood flow

Erythema levels were assessed by reflectance spectrophotometry (Mexameter; Courage – Khazaha) and expressed as arbitrary units (AU, range: ‐ 999). For measurement of mean flux of lesional cutaneous blood circulation laser‐speckle imaging by Moor FLPI blood flow imager (Moor Instruments Ltd.) was applied. This technique is based on laser Doppler imaging and skin blood flow levels are expressed in perfusion units (PU).

### 2.5 Assessment of skin cytokine concentrations

The D‐Squame® technique was used for sampling of lesional corneocytes followed by quantification of inflammasome‐mediated cytokine concentrations. From each skin lesion, 30 consecutive tape strips (D104 ‐ D‐Squame® Standard Sampling Discs, Clinical and Derm) were sampled. Only the last three tape strips, representing the upper epidermal layers were sampled in tubes (2 ml Eppendorf tubes) containing 100 μl of a solution with RIPA buffer (10%, Ref 06/2011 #9806S, Cell signaling technology), protease inhibitor cocktail (10%, #11697498001, Roche Diagnostics GmbH) and distilled water 80%. After 1 h of room temperature incubation with gentle agitation, the samples were stored at −20°C until further use. Before measurement, samples were briefly centrifuged and the three supernatants per subject and treatment were pooled. Levels of cytokines IL‐1β and IL‐18 were measured in duplicate by ELISA (human IL‐1β DuoSet DY201, human IL‐18 DuoSet DY318‐05, R&D Systems) and assessed in pg/ml.

### 2.6 Data and statistical analyses

Statistical analysis was performed for non‐parametric data by using the Friedman‐Test. The IC50 of primary hits was determined by fitting the data to a four parameter logistic equation (Nonlinear Regression, Sigmoidal, 4PL) in GraphPad Prism as described previously.^15^ For all analyses, SPSS version 22.0 and Graph Pad Prism version 6.0 were used. A *p*‐value of ≤0.05 was considered to indicate statistical significance.

## 3 RESULTS

### 3.1 High content screen identifies Disulfiram as a potent NLRP3 inflammasome pathway inhibitor in murine macrophages

In the primary high content screen of 41,184 small molecules, we selected a total of 352 inhibitory compounds via their capacity to inhibit inflammasome activation by assessment ATP‐induced ASC‐speck formation in the murine macrophage reporter cell line and exclusion of cytotoxic and auto‐fluorescent compounds.

In the following selectivity screen, we observed a significant inhibition of ASC speck formation for 56 molecules in the three conditions ATP‐ and nigericin‐induced inflammasome activation as well as inhibition of IL‐ 1β production of ATP and LPS‐treated murine macrophages. Subsequent dose/response assays with 11 different concentrations (20–0.02 μM) for all three conditions narrowed the list down to 48 compounds. Based on the chemical structure, potential for chemical optimization and cell count stability, we selected 10 compounds as candidates of interest. Among those eligible compounds was the thiuramdisulfide‐derived drug disulfiram. Disulfiram (C_10_H_20_N_2_S_4_) is licensed for chronic alcoholism (Antabuse®) as an oral drug.

**FIGURE 1:**
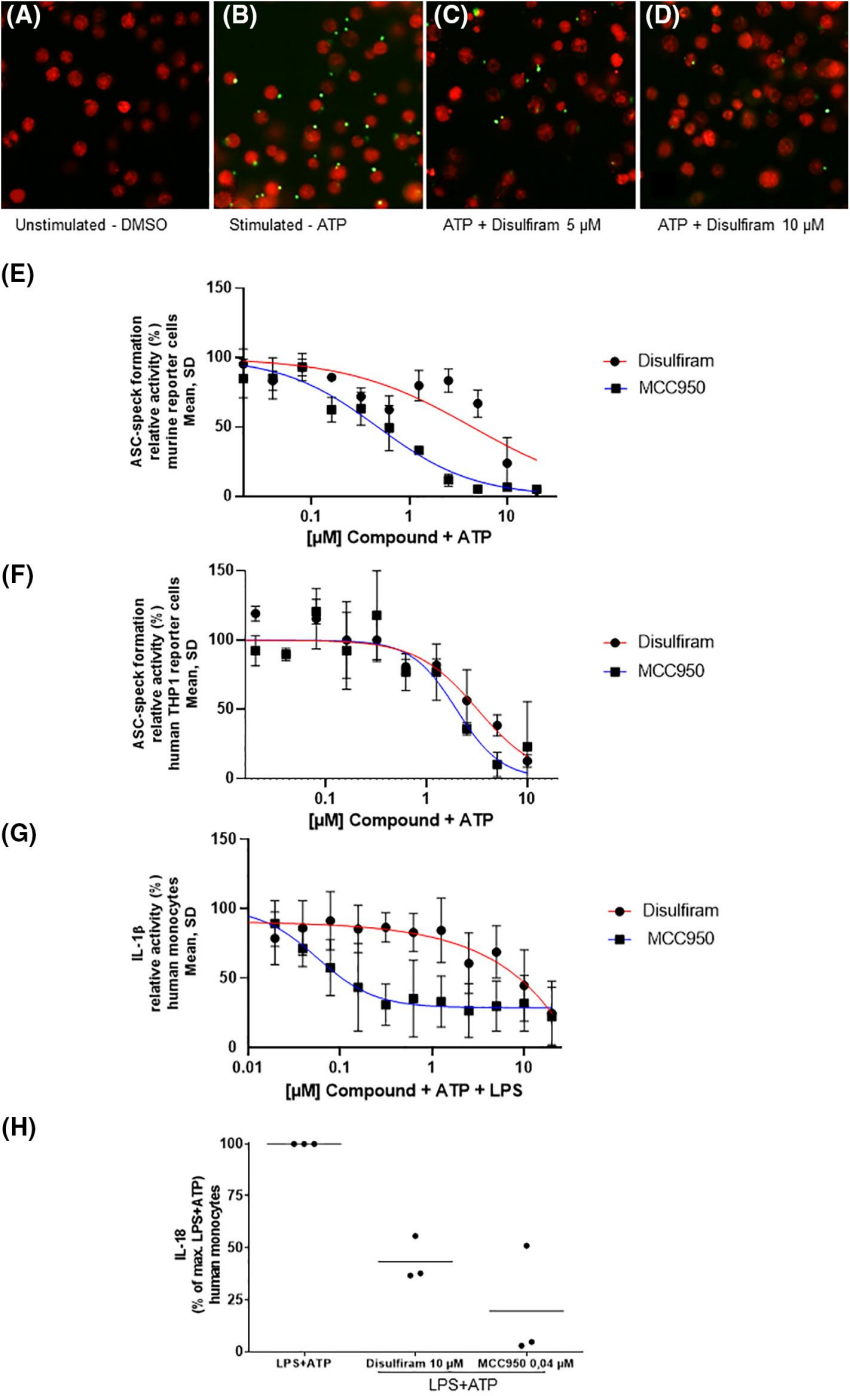
**Disulfiram induced a dose‐dependent inhibition of ASC speck formation and cytokine release in cellular assays** in vitro**.** Fluorescence images showing (A) single nuclei of the murine macrophage reporter cell line without activation vs. (B) activated murine macrophage reporter cells upon Adenosine triphosphate (ATP) stimulation with subsequent Apoptosis associated speck‐like protein containing a CARD (ASC) speck formation (green ASC‐mCerulean) as an indicator of inflammasome activity. Inhibition of ATP‐induced ASC speck formation by (C) Disulfiram 5 μM and (D) 10 μM. Nuclei are counterstained with DRAQ5 (red) (Original magnification x20) (E) Dose‐dependent inhibition of ATP‐induced ASC‐speck formation (relative activity in % in relation to maximal ASC‐formation achieved by ATP‐stimulation of controls) by disulfiram (red curve) in the murine macrophage reporter cell line. MCC950 served as reference substance (blue curve). The results show the data of three replicates. Mean and standard deviation (SD) are shown for each value. (F) Disulfiram inhibits ATP‐induced ASC speck formation in human THP1 ASC‐GFP reporter monocytes dose‐dependently (red curve). MCC950 served as reference substance (blue curve). The results show the data of three replicates. Mean and SD are shown for each value. (G) Dose‐dependent inhibition of Interleukin (IL)‐1β release by disulfiram in human monocytes which were stimulated with ATP and Lipopolysaccharides (LPS) (red curve) compared to reference substance MCC950 (blue curve). IL‐1β release shown as the proportion of the LPS‐ and ATP‐induced release of monocytes in absence of Disulfiram. Mean and standard error of the mean are shown for each value. The graphic shows the results of seven independent experiments. (H) Inhibition of IL‐18 release by disulfiram. Culture supernatants of human monocytes stimulated with LPS + ATP in the presence or absence of disulfiram or MCC950 from three different donors were analyzed by cytokine bead arrays. Relative IL‐18 release (%) in presence of compound as compared to positive control (LPS + ATP) is shown. Bars indicate mean values. The concentrations of disulfiram (10 μM) and MCC950 (0.04 μM) for this assay were selected according to the molar range of their respective IC‐50 derived from the IL‐1β assay on monocytes

Disulfiram inhibited the ATP‐induced ASC speck formation in the murine macrophage reporter cell line dose‐dependently with an IC50 of 4.39 μM. In comparison, the IC50 of the other nine candidates of interest ranged between 0.5 and 7.5 μM (for further information, please refer to Supporting Information S1) while the reference substance MCC950 had an IC50 of 0.47 μM (Figure 1A–E **)**. Disulfiram was selected from the 10 hit compounds due to its approval for human use and its previous application in nickel‐induced allergic contact dermatitis.

### 3.2 Disulfiram dose‐dependently inhibits ASC speck formation and cytokine secretion from human monocytes

Analogously to the murine reporter cell screening and IC50 delineation process, IC50 values of hit compounds were also assessed in human cells using THP1 ASC‐GFP reporter monocytes for the assessment of ASC speck formation and human primary monocytes for inhibition of IL‐1β and IL‐18 secretion.

In line with the murine data, we could observe a dose‐dependent inhibition of ATP‐induced ASC speck formation by disulfiram in the human THP1 ASC‐GFP reporter monocytes (Figure 1F). Here, the IC50 of disulfiram was 3.19 μM, whereas the IC50 of MCC950 averaged 1.97 μM. Likewise but less strongly, disulfiram dose‐dependently inhibited the ATP‐ and LPS‐induced IL‐1β production in human monocytes with an IC50 of 7.38 μM compared to 0.06 μM for MCC950 (Figure 1G). Besides IL‐1β, disulfiram at 10 μM concentration also markedly reduced the secretion of IL‐18 by 56.6% (SD = 11.0) in human monocytes (Figure 1H).

### 3.3 Topical disulfiram dose‐dependently inhibits SDS‐induced irritant contact dermatitis

As an in vivo first proof of concept, we next studied disulfiram for its potential to inhibit irritant contact dermatitis (induced by SDS) in a human patch test model. Therefore, topical disulfiram in different concentrations (0.5%, 2% and 5% in base cream) and vehicle (base cream) were applied on the volar forearm skin of 16 healthy volunteers for 24 h. Afterward, treated skin areas were incubated with SDS 5% for additional 24 h and clinical responses were assessed after further 48 h (Figure S1A).

**FIGURE 2:**
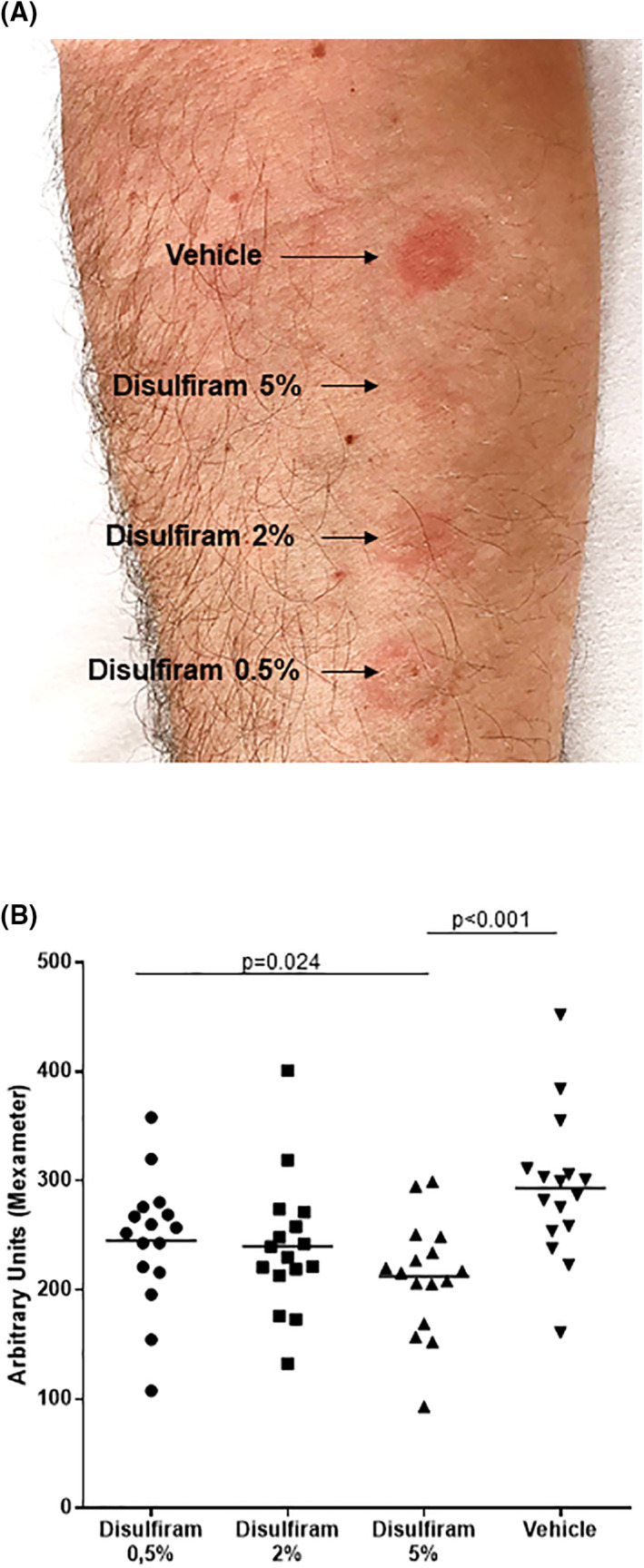
**Topical disulfiram dose‐dependently inhibits SDS‐induced irritant contact dermatitis.** Patch test model. Healthy volunteers were pre‐treated with disulfiram in different concentrations (0.5%, 2% and 5%) or vehicle for 24 h, followed by application of Sodiumdodecylsulfate (SDS) for 24 h. (A) Dose‐dependent inhibition of erythema by pretreatment with disulfiram in base cream. Strong erythema reaction after pretreatment with vehicle, moderated erythema after pretreatment with 0.5% disulfiram, minimal erythema after 2% disulfiram and nearly absence of erythema by pretreatment with 5% disulfiram. Representative example of erythema reaction in one healthy volunteer. (B) Assessment of erythema levels by mexameter in *n* = 16 healthy volunteers of the disulfiram 0.5%, 2% and 5% and vehicle pre‐treated skin. Bars indicate mean values

Here, disulfiram treatment inhibited SDS‐induced inflammation by dose‐dependently reducing erythema levels of skin lesions as compared to vehicle (base cream) (Figure 2). As assessed by reflectance spectrophotometry, erythema levels (mean ± SD) were 28% lower after treatment with 5% disulfiram than with vehicle with significant difference (212.3 ± 52.1 vs. 293.2 ± 66.6 AU). Erythema levels after treatment with 2% and 0.5% disulfiram, respectively, were 239.9 ± 61.5 and 245.0 ± 59.4 AU. The treatment effect achieved with 0.5% disulfiram was significantly less effective as compared to 5% disulfiram but not vehicle (Figure 2B).

### 3.4 Disulfiram is more effective than placebo and at least as effective as mometasone in the treatment of SDS‐induced irritant contact dermatitis

In the second double‐blind randomized controlled part of the study with 25 subjects, topical application of disulfiram 5%, mometasone 0.1% or vehicle (base cream) for 24 h on the volar forearm was followed by application of sodiumdodecylsulfate 5% (SDS) on treated skin areas for 24 h each and clinical responses were assessed after further 48 h.

**FIGURE 3:**
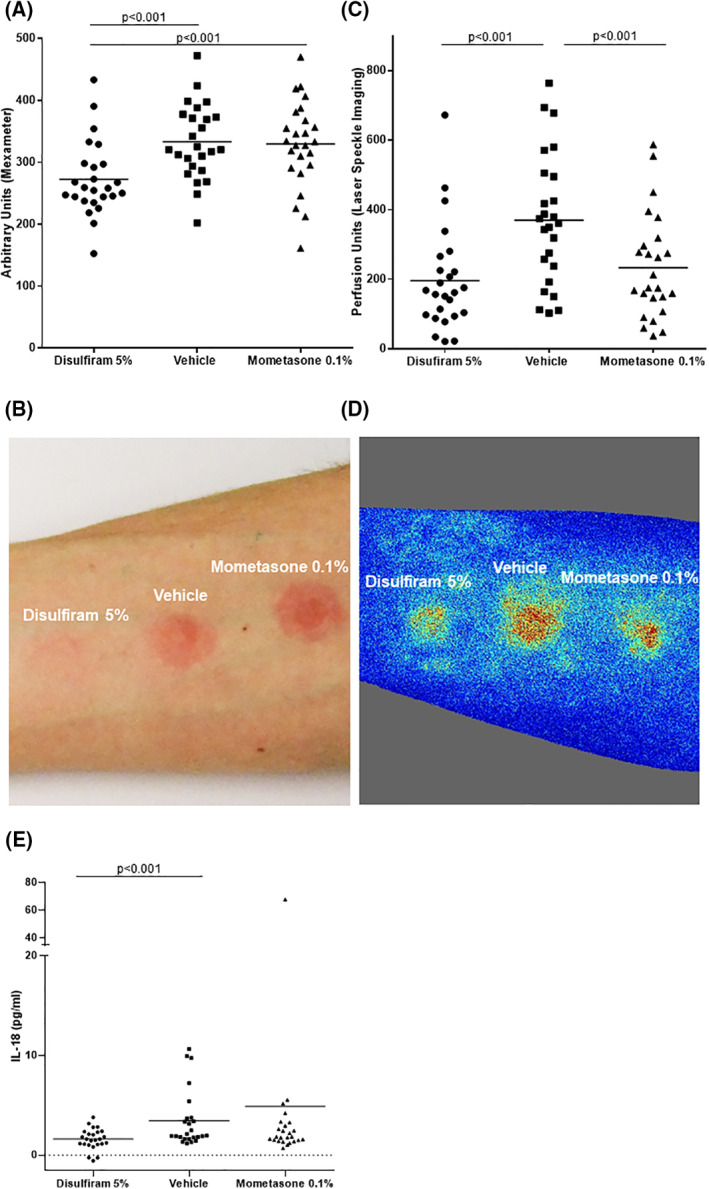
**Disulfiram is more effective than placebo and at least as effective as mometasone in the treatment of SDS‐induced irritant contact dermatitis.** Double‐blind, placebo‐controlled pathophysiology study in *n* = 25 healthy volunteers: Disulfiram inhibits SDS‐induced irritant contact dermatitis. (A) Assessment of erythema by mexameter in *n* = 25 healthy volunteers of the disulfiram 5%, vehicle and momentasone furoate 0.1% pre‐treated skin. Significant reduction of erythema in disulfiram 5% pretreated healthy volunteers compared to mometasone furoate 0.1% treatment and vehicle. Bars indicate mean values. (B) Exemplified clinical picture of erythema reaction in one healthy volunteer. (C) Assessment of perfusion by Laser Speckle Imaging in *n* = 25 healthy volunteers of the disulfiram 5%, vehicle and mometasone furoate 0.1% pre‐treated skin. Significant reduction of blood flow in disulfiram 5% pretreated healthy volunteers compared to vehicle treated group. Bars indicate mean values. (D) Representative example of blood flow assessment by Laser Speckle Imaging in one healthy volunteer. (E) Interleukin (IL)‐18 levels in disulfiram, mometasone furoate 0.1% and vehicle pretreated groups measured in pg/ml by ELISA. Significant reduction of corneocyte IL‐18 levels in disulfiram 5% pretreated healthy controls compared to vehicle. Bars indicate mean values

Topical treatment with disulfiram 5% significantly reduced SDS‐induced irritant contact dermatitis as compared to placebo (Figure 3). Erythema levels of skin lesions (mean ± SD), assessed by reflectance spectrophotometry, were 18% lower after treatment with disulfiram than with placebo with significant difference (272.5 ± 60.0 vs. 333.3 ± 60.4 AU). Also, blood flow in SDS‐induced skin lesions was significantly reduced by 47% as measured by laser speckle imaging (disulfiram: 195.8 ± 150.2 PU; placebo: 369.8 ± 188.2 PU).

Treatment with the class III glucocorticosteroid mometasone 0.1% significantly reduced blood flow compared to placebo (233.1 ± 150.1 PU; −37%), but not erythema levels in SDS‐induced skin lesions (329.6 ± 69.9; −1%). Disulfiram was as effective as mometasone in reducing blood flow and significantly more effective in reducing erythema levels in SDS‐induced skin inflammation (Figure 3A–D).

The topical application of all compounds was well tolerated by the participants. During the study, neither side effects nor allergic skin reactions were reported. The subsequent resolution of eczematous skin lesions was unremarkable in all participants.

### 3.5 Treatment with disulfiram reduces corneocyte cytokine levels in SDS‐induced irritant contact dermatitis.

Corneocyte concentrations of the inflammasome‐mediated cytokines IL‐1β and IL‐18 in treated skin lesions (disulfiram 5%, mometasone 0.1% or vehicle) of SDS‐induced irritant contact dermatitis were assessed by tape stripping and ELISA 48 h after application of SDS. Treatment with disulfiram 5% resulted in significantly lower IL‐18 levels (1.6 ± 1.1 pg/ml) as compared to placebo (3.5 ± 2.9 pg/ml; −54%) but not as compared to mometasone (4.9 ± 13.2 pg/ml) (Figure 3E).

Cutaneous IL‐1β concentrations were very low and not detectable in most samples. Therefore, no significant effects were observed between the three different treatments (data not shown).

## 4 DISCUSSION

Our study shows that disulfiram is a potent and dose‐dependent inhibitor of the NLRP3 inflammasome pathway and that topical treatment with disulfiram is effective in inhibiting irritant contact dermatitis. These findings support the development of inflammasome‐targeted therapies for patients with contact dermatitis such as chronic hand eczema and should prompt further trials to explore the therapeutic potential of topical disulfiram in this disease.

Since the introduction of large‐scale screening technologies in drug discovery, a number of novel drug candidates but also approved drugs with high potential for repurposing were identified. These included compounds for genetic, neoplastic or infective diseases.^16^, ^17^, ^18^

The use of an inflammasome reporter cell line for high content screen is an established method and ATP‐induced ASC speck formation was formerly shown by us to correlate well with caspase‐1 cleavage and IL‐1β production.^12^ Compared to the reference substance MCC950, disulfiram revealed a dose‐dependent, although less potent, inhibition of NLRP3 inflammasome activation in murine and human cellular assays. The superiority of MCC950 compared to disulfiram was highest in the suppression of IL‐1β production in human monocytes. One explanation for these differences could be different modes of action of MCC950 and disulfiram. While MCC950 was shown to directly block canonical and non‐canonical NLRP3 inflammasome activation, disulfiram’s mode of action is less defined and rather considered to be an indirect effect.^19^ For MCC950, as in our study, differences in IC50 levels between ASC speck formation and IL‐1ß release were previously reported. This was explained by the nature of the ASC reporter cell line, which does not require LPS priming owing to its constantly high NLRP3 expression.^14^ Although MCC950 demonstrated to be effective in different inflammatory disorders, it was not further developed due to hepatotoxicity in patients with rheumatoid arthritis during phase II clinical trials.^20^

In allergic or irritant contact dermatitis, NLRP3 inflammasome activation is induced by allergens and irritants respectively, and this results in cutaneous secretion of pro‐inflammatory cytokines IL‐1β and IL‐18.^8^, ^9^, ^10^, ^11^ To assess the potential of disulfiram to inhibit inflammasome pathway activation in vivo, we established a patch test model of irritant contact dermatitis. The different disulfiram concentrations (0.5%, 2% and 5%) were in part derived from an approved antiscabiotic agent (Tenutex®, disulfiram 2%) and anti‐glaucoma eye drops (disulfiram 0.5%).^21^, ^22^ SDS, also known as sodium lauryl sulfate (SLS), is a surfactant molecule that results in lipid extraction and swelling of the exposed skin.^23^ Recently, SDS was shown to induce NLRP3 inflammasome activation in keratinocytes and macrophages.^8^ SDS is an obligatory irritant to the skin and is routinely used in 0.25% concentration to assess skin irritability in patients undergoing patch testing.^24^ In addition, SDS in concentrations up to 10% is applied as a chemical irritant in percutaneous absorption and irritant contact dermatitis models in human skin in vivo.^23^

SDS‐induced eczema models were formerly used to assess the anti‐inflammatory potential of several topically applied herbal substances.^25^, ^26^, ^27^, ^28^ Protective effects of Isatis tinctoria L, marigold, rosemary and Poria cocos were observed when applied in parallel with SDS during the eczema induction phase.^25^, ^26^, ^27^ Comparison to standard glucocorticosteroid treatment was done in one study only without any superiority of the herbal compounds.^26^ Our data are partially consistent with earlier findings showing reduced inflammatory responses in SDS‐induced eczema pre‐treated with topical glucocorticosteroids.^29^

In agreement with the anti‐inflammatory effects of topical disulfiram in our study, systemic treatment with disulfiram previously showed to be effective in patients with active, nickel‐induced allergic contact dermatitis. Altogether three studies with oral disulfiram (two placebo‐controlled trials and one open‐label trial) were performed. Disulfiram doses ranged from 125 to 250 mg/d and treatment duration varied between 4 and 8 weeks. Outcomes showed higher complete responder rates for disulfiram in both placebo‐controlled trials (45% vs. 15% and 91% vs. 10%) and significant clinical improvement in the majority of patients in the open‐label study.^30^, ^31^, ^32^ Notably, hepatotoxicity was observed in single patients treated with disulfiram for hand eczema.^30^, ^31^ In line with this, disulfiram‐induced liver toxicity is the most common side effect in patients with alcoholism, for which disulfiram was originally approved based on its inhibitory effect on the acetaldehyde dehydrogenase. Due to further side effects such as neuropathy and possible embryotoxicity, systemic disulfiram treatment is less frequently used nowadays.

In our patch test model, disulfiram was in part superior in preventing inflammation as compared to mometasone. In contrast to glucocorticosteroids, no broad immunosuppressive effects, skin atrophy or telangiectasia are known from disulfiram usage.^33^

Its topical use as prescription free medication for whole body use in any age is licensed in Sweden and well tolerated.^21^ Besides the use of emollients and avoidance of allergens/irritants, eczema‐preventive treatments are not available. In particular, patients with occupational irritant contact dermatitis could benefit from a protective effect by pro‐active application of disulfiram, thus positively affecting the patients’ working ability and quality of life. Although the topical application of disulfiram was well tolerated, its repetitive use may bear a risk of sensitization. In construction workers with suspected occupational disease and sensitization to rubber components, disulfiram was a common allergen.^34^ Also, a study on patients with suspected allergic contact dermatitis (*n* = 2260) demonstrated a disulfiram sensitization in 4.8% of the cases.^35^ Therefore, the use and dosage of topical disulfiram on inflamed skin need to be assessed over long‐term use.

The mode of action by which disulfiram exerts its anti‐inflammatory effects is not completely understood. When disulfiram was earlier used in nickel‐induced allergic contact dermatitis, it was assumed to act via nickel chelation.^31^ Recent insight showing inflammasome activation to be a relevant pathomechanism in allergic and irritant contact dermatitis^8^, ^9^, ^10^, ^11^ and the in vitro (IL‐1β and IL‐18) and in vivo (IL‐18) decrease of cytokine levels in our study suggest that disulfiram acts via inhibition of inflammasome pathway activation. Recent data support the use of disulfiram in other autoinflammatory conditions such as gout and peritoneal inflammation.^19^ Disulfiram was shown to inhibit the cytoplasmic release of lysosomal cathepsin B resulting in inactivation of the NLRP3 inflammasome. Furthermore, it reduced NADPH oxidase‐derived ROS production.^19^ Of further interest, disulfiram was found to inhibit gasdermin D pore formation.^36^ Whether or not gasdermin D participates in pro‐inflammatory cell death by pyroptosis in the context of allergic and/or irritant contact dermatitis remains open.

Limitations of our study comprise the considerable variability of the assessed clinical parameters, as well as the lack of data on long‐term and therapeutic use of topical disulfiram on inflamed skin, which would require a phase II clinical trial. Based on the favorable effects of systemic disulfiram treatment in patients with nickel‐induced allergic contact dermatitis, its topical use in active disease represents a promising approach.

In contrast to IL‐18, we could not detect cutaneous IL‐1β levels in healthy volunteers. This may be explained by the rapid local degradation of IL‐1β in the tissue as described in other IL‐1‐mediated disorders.^37^ Accordingly, only few IL‐1β‐expressing cells were previously detected in lesional skin of allergic and irritant contact dermatitis, and these did not change over time (6 to 72 h).^38^ However, it should be noted that only upper epidermal layers were examined in our study and dermal cytokine concentrations were not assessed.

In conclusion, we demonstrate a dose‐dependent anti‐inflammatory effect of disulfiram in an experimental study of irritant contact dermatitis. Our results further encourage to investigate the efficacy and safety of topical disulfiram over long term use in inflammasome‐mediated skin diseases. For this, chronic hand eczema due to allergic and/or irritant contact dermatitis would be an excellent condition to study.

## CONFLICT OF INTEREST

Carolina Vera, Angela Abad‐Perez, Silke Radetzki, Martin Neuenschwander, Edgar Specker, Marc Nazaré, Jens v. Kries, Niklas Amadeus Mahnke, Jörg Scheffel and Stefan Frischbutter have no conflict of interest. Eicke Latz is the co‐founder and consultant to IFM Therapeutics. Hanna Bonnekoh received honoria (advisory board, speaker) from Novartis. Marcus Maurer is or recently was a speaker and/or advisor for and/or has received research funding from Allakos, Alnylam, Amgen, Aralez, ArgenX, AstraZeneca, BioCryst, Blueprint, Celldex, Centogene, CSL Behring, Dyax, FAES, Genentech, GIInnovation, Innate Pharma, Kalvista, Kyowa Kirin, Leo Pharma, Lilly, Menarini, Moxie, Novartis, Pharming, Pharvaris, Roche, Sanofi/Regeneron, Shire/Takeda, ThirdHarmonicBio, UCB, and Uriach. Karoline Krause has received grants from Novartis and Roche and payment for lectures and/or consultancies from Novartis, Roche, and SOBI.

## AUTHOR CONTRIBUTION

Hanna Bonnekoh: Conceptualization, Data curation, Formal analysis, Investigation. Writing ‐ original draft: Silke Radetzki, Martin Neuenschwander, Edgar Specker, Marc Nazaré and Jens v. Kries: Data curation, Formal analysis, Investigation, Methodology, Writing ‐ review and editing. Carolina Vera, Angela Abad‐Perez, Niklas Amadeus Mahnke, Jörg Scheffel and Stefan Frischbutter: Data curation, Investigation, Methodology, Writing ‐ review and editing. Eicke Latz and Marcus Maurer: Data curation, Resources, Writing ‐ review and editing. Karoline Krause: Conceptualization, Formal analysis, Funding acquisition, Methodology, Project administration, Writing ‐ original draft.

## Supporting information

Supplementary Material

## References

[1] Skoet R , Zachariae R , Agner T . Contact dermatitis and quality of life: a structured review of the literature. Br J Dermatol. 2003;149(3):452‐456. 10.1046/j.1365-2133.2003.05601.x 14510974

[2] Diepgen TL , Ofenloch RF , Bruze M , et al. Prevalence of contact allergy in the general population in different European regions. Br J Dermatol. 2016;174(2):319‐329. 10.1111/bjd.14167 26370659

[3] Diepgen TL , Andersen KE , Chosidow O , et al. Guidelines for diagnosis, prevention and treatment of hand eczema. J Dtsch Dermatol Ges. 2015;13(1):e1‐e22. 25763418 10.1111/ddg.12510_1

[4] Marron SE , Tomas‐Aragones L , Navarro‐Lopez J , et al. The psychosocial burden of hand eczema: data from a European dermatological multicentre study. Contact Dermat 2018;78(6):406‐412. 10.1111/cod.12973 29464713

[5] Diepgen TL , Scheidt R , Weisshaar E , John SM , Hieke K . Cost of illness from occupational hand eczema in Germany. Contact Dermat 2013;69(2):99‐106. 10.1111/cod.12038 23869729

[6] Lan J , Song Z , Miao X , et al. Skin damage among healthcare workers managing coronavirus disease‐2019. J Am Acad Dermatol. 2020;82:1215‐1216. 10.1016/j.jaad.2020.03.014 32171808 PMC7194538

[7] Scheinman PL , Vocanson M , Thyssen JP , et al. Contact dermatitis. Nat Rev Dis Primers. 2021;7(1):38. 10.1038/s41572-021-00271-4 34045488

[8] Watanabe H , Gaide O , Pétrilli V , et al. Activation of the IL‐1beta‐processing inflammasome is involved in contact hypersensitivity. J Invest Dermatol. 2007;127(8):1956‐1963. 10.1038/sj.jid.5700819 17429439

[9] Li X , Zhong F . Nickel induces interleukin‐1β secretion via the NLRP3‐ASC‐caspase‐1 pathway. Inflammation. 2014;37(2):457‐466. 10.1007/s10753-013-9759-z 24158569

[10] Kambe N , Nakamura Y , Saito M , Nishikomori R . The inflammasome, an innate immunity guardian, participates in skin urticarial reactions and contact hypersensitivity. Allergol Int. 2010;59(2):105‐113. 10.2332/allergolint.09-RAI-0160 20179416

[11] Adam C , Wohlfarth J , Haußmann M , et al. Allergy‐inducing chromium compounds trigger potent innate immune stimulation via ROS‐dependent inflammasome activation. J Invest Dermatology. 2017;137(2):367‐376. 10.1016/j.jid.2016.10.003 27751866

[12] Stutz A , Horvath GL , Monks BG , Latz E . ASC speck formation as a readout for inflammasome activation. Methods Mol Biol. 2013;1040:91‐101. 10.1007/978-1-62703-523-18 23852599

[13] Lisurek M , Rupp B , Wichard J , et al. Design of chemical libraries with potentially bioactive molecules applying a maximum common substructure concept. Mol Divers. 2010;14(2):401‐408. 10.1007/s11030-009-9187-z 19685275 PMC7089384

[14] Coll RC , Robertson AA , Chae JJ , et al. A small‐molecule inhibitor of the NLRP3 inflammasome for the treatment of inflammatory diseases. Nat Med. 2015;3:248‐255. 10.1038/nm.3806 PMC4392179 25686105

[15] Redondo‐Castro E , Faust D , Fox S , et al. Development of a characterised tool kit for the interrogation of NLRP3 inflammasome‐dependent responses. Sci Rep. 2018;8(1):5667. 10.1038/s41598-018-24029-3 29618797 PMC5884858

[16] Coussens NP , Braisted JC , Peryea T , Sittampalam GS , Simeonov A , Hall MD . Small‐molecule screens: a gateway to cancer therapeutic agents with case studies of food and drug administration‐approved drugs. Pharmacol Rev. 2017;69(4):479‐496. 10.1124/pr.117.013755 28931623 PMC5612261

[17] Markossian S , Ang KK , Wilson CG , Arkin MR . Small‐molecule screening for genetic diseases. Annu Rev Genomics Hum Genet. 2018;19:263‐288. 10.1146/annurev-genom-083117-021452 29799800

[18] Fraietta I , Gasparri F . The development of high‐content screening (HCS) technology and its importance to drug discovery. Expet Opin Drug Discov. 2016;11(5):501‐514. 10.1517/17460441.2016.1165203 26971542

[19] Deng W , Yang Z , Yue H , Ou Y , Hu W , Sun P . Disulfiram suppresses NLRP3 inflammasome activation to treat peritoneal and gouty inflammation. Free Radic Biol Med. 2020;152:8‐17. 10.1016/j.freeradbiomed.2020.03.007 32151746

[20] Mangan MSJ , Olhava EJ , Roush WR , Seidel HM , Glick GD , Latz E . Targeting the NLRP3 inflammasome in inflammatory diseases. Nat Rev Drug Discov. 2018;17(8):588‐606. 10.1038/nrd.2018.97 30026524

[21] Landegren J , Borglund E , Storgårds K . Treatment of scabies with disulfiram and benzyl benzoate emulsion: a controlled study. Acta Derm Venereol. 1979;59(3):274‐276. 87094

[22] Kanai K , Ito Y , Nagai N , et al. Effects of instillation of eyedrops containing disulfiram and hydroxypropyl‐β‐cyclodextrin inclusion complex on endotoxin‐induced uveitis in rats. Curr Eye Res. 2012;37(2):124‐131. 10.3109/02713683.2011.622853 22029776

[23] Chiang A , Tudela E , Maibach HI . Percutaneous absorption in diseased skin: an overview. J Appl Toxicol. 2012;32(8):537‐563. 10.1002/jat.1773 22912973

[24] Schnuch A , Aberer W , Agathos M , et al. Performing patch testing with contact allergens. J Dtsch Dermatol Ges. 2008;6(9):770‐775. 10.1111/j.1610-0387.2008.06787.x 19000065

[25] Heinemann C , Schliemann‐Willers S , Oberthür C , Hamburger M , Elsner P . Prevention of experimentally induced irritant contact dermatitis by extracts of Isatis tinctoria compared to pure tryptanthrin and its impact on UVB‐induced erythema. Planta Med. 2004;70(5):385‐390. 10.1055/s-2004-818963 15124080

[26] Fuchs SM , Schliemann‐Willers S , Fischer TW , Elsner P . Protective effects of different marigold (Calendula officinalis L.) and rosemary cream preparations against sodium‐lauryl‐sulfate‐induced irritant contact dermatitis. Skin Pharmacol Physiol. 2005;18(4):195‐200. 10.1159/000085865 15908760

[27] Fuchs SM , Heinemann C , Schliemann‐Willers S , Härtl H , Fluhr JW , Elsner P . Assessment of anti‐inflammatory activity of Poria cocos in sodium lauryl sulphate‐induced irritant contact dermatitis. Skin Res Technol. 2006;12(4):223‐227. 10.1111/j.0909-752X.2006.00168.x 17026651

[28] Zhang YQ , Guan L , Zhong ZY , et al. The anti‐inflammatory effect of cherry blossom extract (Prunus yedoensis) used in soothing skincare product. Int J Cosmet Sci. 2014;36(6):527‐530. 10.1111/ics.12149 25065693

[29] Jungersted JM , Høgh JK , Hellegren LI , Jemec GB , Agner T . Effects of topical corticosteroid and tacrolimus on ceramides and irritancy to sodium lauryl sulphate in healthy skin. Acta dermato‐venereologica. 2011;91(3):290‐294. 10.2340/00015555-1064 21365172

[30] Kaaber K , Menné T , Veien N , Hougaard P . Treatment of Nickel dermatitis with Antabuse; a double blind study. Contact Dermat 1983;9(4):297‐299. 10.1111/j.1600-0536.1983.tb04394.x 6352169

[31] Christensen OB , Kristensen M . Treatment with disulfiram in Chronic Nickel hand dermatitis. Contact Dermatitis. 1982;8(1):59‐63. 7067441 10.1111/j.1600-0536.1982.tb04137.x

[32] Sharma AD . Disulfiram and low nickel diet in the management of hand eczema: a clinical study. Indian J Dermatol Venereol Leprol. 2006;72(2):113‐118. 16707816 10.4103/0378-6323.25635

[33] The MAK‐Collection for Occupational Health and Safety. 1997.

[34] Condé‐Salazar L , Guimaraens D , Villegas C , Romero A , Gonzalez MA . Occupational allergic contact dermatitis in construction workers. Contact Dermatitis. 1995;33(4):226‐230. 10.1111/j.1600-0536.1995.tb00471.x 8654071

[35] Geier J , Gefeller O . Sensitivity of patch tests with rubber mixes: results of the information network of departments of dermatology from 1990 to 1993. Am J Contact Dermat. 1995;6:143‐149.

[36] Hu JJ , Liu X , Xia S , et al. FDA‐approved disulfiram inhibits pyroptosis by blocking gasdermin D pore formation. Nat Immunol. 2020;21(7):736‐745. 10.1038/s41590-020-0669-6 32367036 PMC7316630

[37] Lachmann HJ , Lowe P , Felix SD , et al. In vivo regulation of interleukin 1beta in patients with cryopyrin‐associated periodic syndromes. J Exp Med. 2009;5:1029‐1036. 10.1084/jem.20082481 PMC2715040 19364880

[38] Ulfgren AK , Klareskog L , Lindberg M . An immunohistochemical analysis of cytokine expression in allergic and irritant contact dermatitis. Acta dermato‐venereologica. 2000;80(3):167‐170. 10.1080/000155500750042899 10954204

[39] Bland JM , Altman DG . Statistical methods for assessing agreement between two methods of clinical measurement. Lancet. 1986;1(8476);307‐310. 2868172

