# Supplementary material for: Topical inflammasome inhibition with disulfiram prevents irritant contact dermatitis
Source: Clin Transl Allergy. 2021 Jul 22;11(5):e12045. doi: 10.1002/clt2.12045 (PMC8297992; doi:10.1002/clt2.12045)
Supplement: Supplementary file 1 — Supplementary Material [file CLT2-11-e12045-s001.docx]

**Appendix**

**Supplementary Material**

**1**

We used a murine macrophage reporter cell line (ASC-mCerulean NLRP3 flag N3ko [clone 19.5] Institute of Innate Immunity, University of Bonn, Germany) expressing a fluorescently tagged apoptosis associated speck-like protein containing a CARD (ASC) protein (ASC-mCerulean). The reporter cell line was cultured in DMEM (Dulbeccos's modified eagle medium, Thermofisher, Massachusetts, USA) with 10% FBS (Fetal bovine serum, Biochrom AG, Berlin, Germany) and 1% penicillin/streptomycin (Biochrom AG, Berlin, Germany) in adhesion cell culture flasks (175 cm^2^, Sarstedt, Nümbrecht, Germany). When a confluence of 80% was reached, cells were washed with PBS (Phosphate-buffered saline, 14190094 Thermo Fisher Scientific, Hennigsdorf, Germany), seeded with 8000-10 000 cells/ per well in 30 µl volume into 384-well microplates (CellBIND, Corning Life Sciences, Acton, MA, USA) and incubated at 37°C overnight. Subsequently, the cells were incubated with the compounds either at 10 µM or at different concentrations (each 0.02, 0.04, 0.08, 0.16, 0.3, 0.6, 1.25, 2.5, 5, 10, 20 µM) for 1h, followed by additional 10 µl per well of NLRP3-inflammasome activators either 2.5 mM ATP (primary screen and selectivity screen; Sigma Aldrich, Taufkirchen, Germany) and/or 13.8 µM nigericin (selectivity screen only; CAS 28643-80-3, Invivogen, San Diego, California) and incubation for 1h at 37°C. The known inflammasome inhibitor MCC950^14^ (CAS 210826-40-7 Selleck Chemicals, Munich, Germany; Tocris Bioscience, Bristol, UK) served as reference substance. Afterwards, 25 µl of 8% paraformaldehyde (PFA) was added to each well for cell fixation. Nuclei were stained with 2 µM DRAQ5^TM^ (BioStatus, Leicestershire, United Kingdom) and plates were stored at 4°C.

ASC is expressed in the cytoplasm ubiquitously. Upon activation by stimuli such as ATP or nigericin, ASC forms small particles called specks, indicating NLRP3 inflammasome activation. The ASC speck formation was visualized and assessed by automated fluorescence microscopy, 20x magnification. An Array-Scan XTI Reader (Thermo Fisher Scientific Inc.) was used to acquire images in the DRAQ5 –associated filter channel (BGRFR 650_13) for nuclei detection and the mCerulean-associated filter channel (BGRFR 485_20) for the ASC speck detection with a 20x objective. For each well, 9 image fields were acquired, ensuring that typically 3000 cells or more could be analyzed using the Spot Detector analysis bioapplication of the HCS Studio software. The number of ASC specks inside a defined circle (10 µm around nuclei) was determined for the calculation of speck number per cell as readout parameter. Valid specks were identified applying thresholds for size and intensity. The compound activity was calculated relative to the maximum activity of 100% by ATP induction. Each measured 384-well plate contained 352 compounds samples in columns 1-22, 14 DMSO-control samples in column 23 rows A-N, 14 no-ATP induction control samples in column 24 rows A-N, and 4 reference compound control samples in rows O and P and columns 23 and 24 that were incubated with 10 µM of the control compound MCC950. 3 parameters were used for data analysis: the nuclei count per well in the fluorescence channel used for nuclei detection, the average fluorescence intensity around the detected nuclei in the fluorescence channel used for speck detection, and the total number of specks detected in each well. To assess data quality, the effective signal window (Z’-factor) was calculated for each plate for the parameter total number of specks, using the DMSO- and no-ATP induction control samples.

The data was then normalized for each plate by calculating the z-score value for each sample well for all 3 parameters (where the z-score describes the distance of the signal of a specific well from the mean of signals of all sample wells in a plate, in units of standard deviation). For the selection of potential inflammasome inhibitors, the z-score values were used. A sample was considered active for inhibition of ASC speck formation when the z-score for the total number of ASC specks was smaller than -3. A sample was considered being toxic when the z-score for the nuclei count was smaller than -4, and being autofluorescent if the z-score for the average fluorescence intensity was larger than 4. Samples were selected as inhibitors that were active in reduction of speckle formation, but that were not toxic or auto-fluorescent. Instead of using the z-score value, PercentActivity values were calculated for each sample on a plate by comparing the value to the DMSO-control samples (giving the reference value for 100% speckle formation) and no-ATP induction control samples (giving the reference value 0% speckle formation). Measurements were performed multiple technical replicates, and reproducible activity was assessed by taking the data of 2 technical replicates and displaying the PercentActivity values on a mean-difference plot (Bland-Altman plot).^39^ Limits for activity determination for each condition were estimated visually from the Bland-Altman diagrams. The supernatants of the ATP plus lipopolysaccharides (LPS)-induced 384-well microtiter plates were used to assess IL-1β production by ELISA.

For the selectivity screen, resulting substances from the primary screen were identified and subsequently re-examined with inflammasome activators like ATP as well as nigericin (Cayman Chemical, Michigan, USA) or stimulation with ATP after priming with LPS (E.coli Serotype R515, TLR quality, R-form, Enzo Life Sciences, Lörrach, Germany) in the murine ASC reporter cell line. This was followed by assessment of ASC speck formation by microscopic analysis. For the LPS and ATP stimulation of the murine cell line, the secretion of IL-1β (LPS and ATP) was also measured by ELISA (affymetrix eBioscience: Mouse IL-1 beta ELISA Ready-SET-Go!). The final selection for the following IC50 validation was made by limiting the substances to those that had both inhibitory effect after stimulation with ATP, ATP and LPS or nigericin in the fluorescence microscopy, as well as in IL-1β ELISA inhibitory activity after ATP and LPS stimulation. The identified inhibitory substances from the selectivity screen were assessed in inhibitory concentration (IC)-50 validation (High Content Dose Response Assay with 11 concentrations from 20 µM to 0.02 µM).

**2**

To corroborate the results of the selectivity screen, ASC speck formation was visualized and assessed by automated fluorescence microscopy using human THP1 ASC-GFP reporter monocytes (Invivogen® San Diego, California). The reporter cells were cultured following suppliers instructions in RPMI 1640 (Biochrom, Berlin, Germany) with 10% FBS, 1% penicillin/streptomycin and 50 mg/ml Normocin® (Invivogen, San Diego, California, USA) in adhesion cell culture flasks (175 cm^2^, Sarstedt, Nümbrecht, Germany) with confluence of 5*10^6^ cells/ml. Every second cycle, Zeocin© 100 mg/ml (Invivogen, San Diego, California, USA) was added. 8000-10 000 cells/ per well were seeded into 384well clear bottom microplates overnight at 37°C, followed by incubation with the compounds at eleven concentrations (each 0.02, 0.04, 0.08, 0.16, 0.3, 0.6, 1.25, 2.5, 5, 10, 20 µM) and additional 10 µl per well NLRP3 activator 2 mM ATP and incubation for 1h at 37 C°. Further processing was analogous to the murine cell line (see above).

The selectivity screen resulted in the identification of 10 hit compounds including disulfiram. The other 9 compounds comprised known drugs such as auranofin, a VEGFR tyrosin kinase, a STAT3 inhibitor and other novel chemical entities with different chemical structures.

Additionally, we evaluated the IL-1β and IL-18 release of human primary monocytes after 2 µM ATP and 0.5 ng/ml LPS stimulation in the presence of the identified compounds. Briefly, peripheral blood mononuclear cells were isolated by using LeucoSep® tubes (Greiner bio-one, Kremsmünster, Austria). Tubes were filled with 15 ml of Ficoll-Paque medium (GE Healthcare, Calfont St Giles, UK) and centrifuged (1000 x g, 30s). 2x9 ml heparin blood (5 U/ml heparin) and 17 ml of PBS were added and then centrifuged (1000 x g without brake, 15 min) again. The cell fraction was harvested and washed with PBS twice (350 x g, 10 min). The resulting pellet was resuspended in culture medium (RPMI 1640 with 1% FBS and 1% penicillin/streptomycin) to a final concentration of 2.7*10^6^ cells/ml. To obtain monocytes, the cells were seeded in 96 well adhesion cell culture plates (270 000 cells/well) in 100 µl culture medium. After 2h of incubation (37°C, 5% CO_2_), the medium containing non-adherent cells was removed and adherent were carefully washed with medium and used for further experiments. Fresh culture medium with 0.5 ng/ml LPS was added for overnight stimulation. After LPS-priming, culture medium was again changed and compounds were added in either 10 µM or for IC50 in eleven concentrations (20-0.02 µM) for 1h. Afterwards, cells were stimulated with 2 µM ATP for 1h. Subsequently, IL-1β release in the supernatants was assessed by ELISA (human IL-1β DuoSet DY201, R&D Systems, Minneapolis, Minnesota). All compounds were tested in duplicates at 10 µM. All dose responses were tested using monocytes from 7 healthy volunteers in independent experiments. To quantify the secretion of IL-18 by human primary monocytes, cell culture supernatants were harvested and cytokine release was measured using a bead based multiplex cytokine assay (Cytokine 25-Plex human ProcartaPlex Panel 1B, Thermo Fisher Scientific) according to the manufacturer’s instructions with the following modification: all samples were incubated with antibody coated beads for 1h at room temperature followed by incubation overnight at 4°C. All samples were analyzed using the Bioplex 200 system (Bio-Rad Laboratories GmbH).

**3**

For in vivo studies on inhibition of inflammasome activation in a model of SDS-induced eczema, disulfiram was applied topically. Disulfiram (United States Pharmacopeia (USP), 1971-05, Euro-Chemicals, Oldenzaal, Netherlands) was mixed with medium-chain triglycerides in equal shares and suspended in base cream (DAC Deutscher Arzneimittelcodex, [ingredients: glycerol monostearate 60, cetyl alcohol, medium-chain triglycerides, white petrolatum, macrogol-1000-glycerol monostearate, propylene glycol, purified water] Fagron, Barsbüttel, Germany) homogenously. This was done for different concentrations (0.5%, 2% and 5%) and the final products were packaged in 100g aluminum tubes and stored at ambient temperature. For the placebo-controlled study part, block randomization was applied. Participants were divided into n=5 blocks consisting of n=5 subjects each. Per week, n=5 participants were allocated to the same order of application of disulfiram 5% in base cream, mometasone furoate 0.1% in base cream and vehicle. The creams were labeled as A, B and C and applied at the inner forearm (**Supplementary Material Figure 1B**). Every week, the allocation of the three creams to the labeling A, B or C was alternated. All subjects received the same topical treatment.

Healthy subjects, physicians and site personnel were blinded to the topical treatment assignment. To preserve blinding, mometasone furoate 0.1% in base cream, disulfiram 5% in base cream and base cream as vehicle were packaged identically. One unblinded physician prepared the allocation of the three creams to the respective labelling for every week of the study.

**Supplementary Figure:**

**Figure S1:** Overview on the study procedures and timeline for the **A)** unblinded proof of concept dose-response study in 16 healthy volunteers receiving 0.5%, 2% and 5% disulfiram in base cream versus vehicle in SDS-induced contact eczema on the forearm and **B)** the double-blind and placebo-controlled experimental study in 25 healthy volunteers on 5% disulfiram in base cream, mometasone furoate 0.1% in base cream and the vehicle in SDS-induced contact eczema.
